# N-myristoylation of Antimicrobial Peptide CM4 Enhances Its Anticancer Activity by Interacting With Cell Membrane and Targeting Mitochondria in Breast Cancer Cells

**DOI:** 10.3389/fphar.2018.01297

**Published:** 2018-11-13

**Authors:** Caiyun Li, Hongyan Liu, Yunqing Yang, Xixi Xu, Tongtong Lv, Huidan Zhang, Kehang Liu, Shuangquan Zhang, Yuqing Chen

**Affiliations:** Jiangsu Key Laboratory for Molecular and Medical Biotechnology, Life Sciences College, Nanjing Normal University, Nanjing, China

**Keywords:** antimicrobial peptides CM4, myristoylation, breast cancer, membrane, mitochondria, xenograft tumor

## Abstract

Development of antimicrobial peptides (AMPs) as highly effective and selective anticancer agents would represent great progress in cancer treatment. Here we show that myristoyl-CM4, a new synthetic analog generated by N-myristoylation of AMPs CM4, had anticancer activity against MCF-7, MDA-MB-231, MX-1 breast cancer cells (IC_50_ of 3–6 μM) and MDA-MB-231 xenograft tumors. The improved activity was attributed to the effect of myristoyl on the cell membrane. Flow cytometry and confocal laser scanning microscopy results showed that N-myristoylation significantly increased the membrane affinity toward breast cancer cells and also effectively mediated cellular entry. Despite increasing cytotoxicity against HEK293 and NIH3T3 cells and erythrocytes associated with its anticancer activity, myristoyl-CM4 maintained a certain selectivity toward breast cancer cells. Accordingly, the membrane affinity toward breast cancer cells was two to threefold higher than that of normal cells. Glycosylation analysis showed that sialic acid-containing oligosaccharides (including *O*-mucin and gangliosides) were important targets for myristoyl-CM4 binding to breast cancer cells. After internalization, co-localization analysis revealed that myristoyl-CM4 targeted mitochondria and induced mitochondrial dysfunction, including alterations in mitochondrial transmembrane potential, reactive oxygen species (ROS) generation and cytochrome c release. Activation of caspase 9, caspase 3 and cleavage of PARP were observed in MX-1, MCF-7, and MDA-MB-231 cells after myristoyl-CM4 treatment. The current work indicates that increasing hydrophobicity by myristoylation to modulate peptide-membrane interactions and then target mitochondria is a good strategy to develop AMPs as anticancer agents in the future.

## Introduction

Cancer is a major public health problem worldwide and the global cancer death will rise to more than 13 million by 2030. In the United States, statistical data from 2017 show that 1,735,350 new cancer cases and 609,640 cancer deaths are projected to occur (Siegel et al., 2018). Breast cancer is the most common cancer and also the primary cause of mortality in female around the world (Akram et al., 2017). Along with surgery as first line treatment, chemotherapy remains the standard treatment in breast cancer. Several strategies including endocrine therapy (ER^+^ or/and PR^+^), HER2^-^ targeted therapy (HER2^+^), BRCA1/BRCA2 therapy against the BRCA1/BRCA2 mutation, PI3K-mTOR targeted therapy, CDK4/6^-^ targeted therapy and DNA-alkylating agents have been developed and are currently used in clinical practice (Mohamed et al., 2013). However, insufficient selectivity and consequently targeting of healthy mammalian cells with many deleterious effects, as well as the development of resistance remain a serious challenge, especially in triple negative patients (Tobin et al., 2015). Therefore, the development of new effective anticancer drugs with low toxicity to normal host cells would represent great progress in the treatment of cancers including breast cancer.

Antimicrobial peptides (AMPs), which are effective components of innate immunity, have emerged as potential alternative anticancer therapeutics with many advantages. Most anticancer AMPs share a common membranolytic mode of action via a non-receptor-mediated pathway, which results in the selective disruption of the cancer cell membrane or permeation of the membrane and an effect on mitochondria (Gaspar et al., 2013; Chen et al., 2014). With regard to cellular targets, anticancer AMPs can be assigned to AMPs that are highly potent against cancer cells but not against healthy mammalian cells, and AMPs that are cytotoxic to both cancer cells and normal mammalian cells. AMPs with cancer-selective toxicity have received much attention as alternative anticancer therapeutics to overcome the limitations of current drugs. However, the successful generation of anticancer AMPs with high anticancer activity and low side-effects is difficult because it relies on the manipulation of many aspects of the molecule, such as sequence, net charge, secondary structure, amphipathicity, and hydrophobicity (Teixeira et al., 2012). Many reported cancer-selective AMPs either have potent anticancer activity with relatively low selectivity or relatively low anticancer activity with high selectivity, which hampers their use in cancer therapy (Lu and Chen, 2010; Zhang et al., 2010). Despite numerous anticancer peptides reported in the literature, none of the AMPs has been approved for clinical use to date.

Covalent modification of proteins by fatty acids such as myristate or palmitate is a widely recognized form of protein modification and plays essential roles in directing the cellular localization of proteins by facilitating protein-membrane interactions as protein-protein interactions (Martin et al., 2011). Several studies have examined the efficacy of modifying the N-terminus with different fatty acids to improve the antibacterial activity of AMPs (Avrahami and Shai, 2004; Lockwood et al., 2004; Chu-Kung et al., 2010). The results indicate that modification by fatty acids may change the antimicrobial activity and selectivity of AMPs. However, at least two questions arise, (1) whether the anticancer activity is affected by fatty acid modification, and (2) whether the modification changes the target of AMPs in cancer cells.

Cationic amphiphilic peptide CM4 is an effective AMP against bacteria as well as fungi that functions by affecting membrane interactions (Zhang et al., 2008; Li et al., 2012). It also has anti-inflammatory effects mediated by the neutralization of the endotoxin lipopolysaccharide and anticancer activity in leukemia cells by targeting and disrupting the plasma membrane (Lin et al., 2008; Chen et al., 2010). Even at a concentration of 200 μM, it has no hemolytic activity in human erythrocytes. In the present study, we attached myristoyl, a 14-carbon saturated fatty acid, to the N-terminus of CM4 and investigated its anticancer activity against breast cancer cells *in vitro* and *in vivo*. Myristoyl-CM4 showed significantly enhanced anticancer activity in breast cancer cells and MDA-MB-231 xenograft tumor. The interaction between myristoyl-CM4 and the breast cancer cell membrane was further investigated to explain its anticancer mechanism.

## Materials and Methods

### Reagents

MTT (cat.no. KGA311, KeyGEN Biotech, Nanjing, China), TFE (cat.no. 75898, Sigma–Aldrich, St. Louis, MO, United States), DAPI (cat.no. 17510, FANBO Biochemicals, Beijing, China), Rho123 (cat.no. 83702, Sigma–Aldrich, St. Louis, MO, United States), L-PPMP (cat.no. 149022184, Sigma–Aldrich, United States), BnGalNAc (cat.no. 3554936, Sigma–Aldrich, United States), tunicamycin (cat.no. 654380, Sigma–Aldrich, St. Louis, MO, United States). Sialidase (cat.no. N7885, Sigma–Aldrich, United States) from *Vibrio cholerae*. Hoechst 33342/PI staining kit (cat.no. KGA212, KeyGEN Biotech, Nanjing, China), ROS assay kit (cat.no. S0033, Beyotime Institute of Biotechnology, Shanghai, China), DCFH-DA (cat.no. s0033, Beyotime Institute of Biotechnology, Shanghai, China). JC-1 Apoptosis Detection kit (cat.no. KGA603, KeyGEN Biotech, Nanjing, China), Annexin V/PI assay kit (cat.no. KGA107, KeyGEN Biotech, Nanjing, China). TUNEL Apoptosis Assay Kit (cat.no. 6432344001, Roche, United States), Antibodies against PARP (cat.no. sc-136208), Bax (cat.no. sc-7480), Bcl-2 (cat.no. sc-7382), cytochrome c (cat.no. sc-13561), caspase 9 (cat.no. sc-73548), caspase 3 (cat.no. sc-56053), CD31 (cat.no. sc-376764), PCNA (cat.no. sc-25280), and GAPDH (cat.no. sc-137179) were purchased from Santa Cruz Biotechnology (Santa Cruz, CA, United States). All other reagents were analytical grade reagents, and produced in China. All the reagents were used by the rules of standard bio-security and safety procedures of Nanjing Normal University.

### Cell Lines and Cell Culture

Breast cancer cell lines MCF-7, MX-1 and MDA-MB-231 and normal cell lines human embryonic kidney cells HEK-293 and mouse embryonic fibroblasts cells NIH3T3 were obtained from the Shanghai Institute of Biochemistry and Cell Biology, Chinese Academy of Sciences. All the cell lines used in the experiment were cultured at 37°C, with 5% CO_2_ in a humidified incubator (Forma 3111; Thermo Scientific, Lincoln, NE, United States). MCF-7, HEK-293 and MDA-MB-231 were cultured in DMEM (cat.no. 12491023, Gibco, United States) supplemented with 10% fetal bovine serum (FBS) (cat.no. 26400044, Gibco, United States). MX-1 and NIH3T3 were grown in RPMI-1640 medium (61870044, Gibco, United States). All the media used in the experiment were supplemented with 100 μg/mL streptomycin, 100 U/mL penicillin and 10% FBS.

### Peptide Synthesis

The peptide of CM4 (GRWKIFKKIEKVGQNIRDGIVKAGP AVAVVGQAATI-NH_2_), myristoyl-CM4, FITC-labeled CM4 and FITC-labeled myristoyl-CM4 were synthesized using solid-phase Fmoc methods by Biomatik Corporation (Cambridge, ON, Canada). The synthesized peptides used were all of >95% homogeneous as indicated by C18 reverse-phase HPLC and ESI mass spectroscopy analysis.

### Circular Dichroism (CD) Spectra

Myristoyl-CM4 and CM4 were diluted in 50% TFE and final concentration is 250 μg/mL. Circular Dichroism spectra measurements were performed in a quartz cuvette of 0.1 cm path-length at room temperature. Samples were scanned from 180 to 260 nm at 0.1 nm/min with a Chirascan spectrophotometer (Applied Photophysics). Then the CD spectra data were recorded as mean residue ellipticity ([𝜃]). CDNN program was used to calculate the helical content of each peptide.

### Cell Viability Assay

Cells were cultured in DMEM or RPMI-1640 supplemented with 10% FBS. When the cell density reaches 80%, cells were cultured in 96-well plates at a density of 1 × 10^5^/mL in medium supplemented with 1% FBS, and then treated with different concentrations (2, 4, 8, 16, 32 μM for myristoyl-CM4 and 4, 8, 16, 32, 64 μM for CM4) of the peptides. After treated for 24 h, MTT stock solution was added to each well at a final concentration of 500 μg/mL and incubated in the dark for 4 h. The medium was then removed and DMSO was added to dissolve the formazan, finally, the absorbance at 570 nm (test wavelength) and 630 nm (reference wavelength) using a SynergyTM 2 Multi-function Microplate Reader. Data reported in the figures are the mean ± SEM of 4–6 independent experiments.

### Hemolytic Activity Assay

Erythrocytes were isolated from fresh mouse blood cells by centrifugation at 1000 *g* for 10 minutes (min) and washed three times with PBS. Hemolytic activity was evaluated to the method described previously (Singh et al., 2016). Briefly, erythrocytes (final concentration 4% v/v) were treated with myristoyl-CM4 or CM4 for 1 h at 37°C, followed by centrifugation at 1000 *g* for 5 min. The absorbance of the supernatants was measured at 414 nm. For 100% hemolysis and 0% hemolysis, 0.1% TritonX-100 (v/v) and PBS were used respectively. Melittin, a hemolytic peptide from *bee Apis mellifera* was used as a control. The percentage of hemolysis was calculated as: (A_peptide_-A_PBS_)/(A_TritonX-100_-A_PBS_) × 100%. Data reported in the figures are the mean ± SEM of 4–6 independent experiments.

### Peptide Binding Assay

Cells (1 × 10^5^/mL) were collected and re-suspended in PBS. The binding activities of the peptides were assessed using FITC-myristoyl-CM4 or FITC-CM4. After incubation at 37°C for different times (5, 10, 20, 30 min) in the dark, cells were washed with PBS and then observed by confocal laser scanning microscopy (CLSM) at 488 nm excitation. Cells (2 × 10^5^/mL) were collected and re-suspended in PBS. After incubation with FITC-myristoyl-CM4 or FITC-CM4 at 37°C for 30 min in the dark, cells were washed with PBS and the mean fluorescence of 10000 cells was analyzed with BD flow cytometry software for each sample, the autofluorescence of non-treated cells was subtracted from the data of cells incubated with FITC-CM4 and FITC-myristoyl-CM4. Data reported in the figures are the mean ± SEM of 3 independent experiments.

### Sialidase and Inhibitors Treatments

Cells (MCF-7, MX-1) were seeded in 6-well plates (1 × 10^5^/well) for 12 h at 37°C, then maintained in phenol red-free, FBS-free medium and pretreated as follows: 0.1 U/ml sialidase for 30 min, 2 mM of BnGalNac for 48 h, 3 μg/ml tunicamycin for 24 h, or 2 μM of L-PPMP for 48 h, respectively. After washing with PBS to remove the treatment reagent, the cells were incubated with 2 μM of FITC-myristoyl-CM4 for 30 min. After washing with PBS, the cells were analyzed by flow cytometry at 488 nm excitation. The cells by L-PPMP treatment were also observed by CLSM(excitation, 488 nm; emission, 525 nm).

### Fluorescence Double Staining

Cells at a density of 1 × 10^5^/mL were incubated with 30 nM Rho123 for 45 min in the dark then the cells were washed with PBS and treated with 2 μM of FITC-myristoyl-CM4 for 30 min in the dark. After washing with PBS, the distribution of fluorescence was immediately observed by CLSM. Optical excitation was carried out with a 488 nm argon laser beam for the FITC signal and 525 nm for the Rho123 signal.

### Mitochondrial Membrane Potential (Δψm)

Change in Δψm was detected using a mitochondria staining kit that uses JC-1, a cationic fluorescent dye. Briefly, cells (1 × 10^5^/mL) were seeded into a 6-well plate and exposed to 2, 4, or 8 μM myristoyl-CM4, after treated for 16 h, the dye JC-1 was added at a final concentration of 1 μM for 40 min at room temperature and then washed with the JC-1 washing buffer. Cells were placed on ice until analyzed by flow cytometry. For JC-1 monomers, the flow cytometry was set at 490 nm excitation and 530 nm emission wavelengths, for JC-aggregates, the wavelengths were set at 525 nm excitation and 590 nm emission.

### Detection of ROS Accumulation

Reactive oxygen species accumulation was assayed quantitatively by detecting the fluorescent intensity of oxidant-sensitive probe DCFH-DA as described (Kang and Yan, 2015). Briefly, Cells (MCF-7, MDA-MB-231 and MX-1) were seeded in 6-well plates (1 × 10^5^/well) were incubated with 2, 4, and 8 μM myristoyl-CM4 for 10 h, then the cells loaded with DCFH-DA (10 μM) for 30 min in the dark and then the fluorescence intensity was measured at 488 nm by flow cytometry to evaluate the production of ROS. Rosup was used as positive control.

### Hoechst 33342/PI Staining and Annexin-V-FITC/PI Staining

Cells (1 × 10^5^/mL) were seeded into 6-well plates and treated with myristoyl-CM4 (4 μM for MCF-7, 6 μM for MDA-MB-231, 3 μM for MX-1) for 16 h. Apoptotic nuclei were detected by Hoechst 33342 (v/v at 1:200) and PI (v/v at 1:200) at 37°C for 20 min in the dark. Then washed three times by PBS and acquired images by fluorescence microscopy. Apoptosis was evaluated by double staining with Annexin V-FITC and PI for 10 min on ice in the dark. The stained cells were analyzed by flow cytometry and the percentage of apoptotic cells was calculated using Cell Quest software.

### Western Blotting Analysis

Cells (1 × 10^5^/mL) were seeded into 6-well plates and treated with peptides for 16 h at 37°C. After treatment, cells were lysed in RIPA lysis buffer (cat.no. P0013B, Beyotime institute of Biotechnology, Shanghai, China), and centrifuged at 15000 *g* for 10 min. Supernatants were collected, separated on 12% SDS–PAGE gels and transferred onto PVDF membranes. Membranes were blocked with 5% BSA and probed with polyclonal antibodies against cytochrome c, caspase 9, caspase 3, PARP and GAPDH. Protein bands were visualized using the Odyssey infrared imaging system and analysis by image J densitometric analysis software.

### *In vivo* Tumorigenesis

Female BALB/c nude mice were injected subcutaneously right axillary with MDA-MB-231 cells (1 × 10^6^/mL). When the tumors measured approximately 75 mm^3^, the mice were randomly divided into four groups (five mice per group) and treated as follows: group 1, myristoyl-CM4 at a dose of 5 mg/kg; group 2, cisplatin at a dose of 5 mg/kg and group 3, equivalent volumes of PBS, group 4, CM4 at a dose of 5 mg/kg. All compounds were injected through the tail vein of mice on days 1, 2, 4, 6, and 8. Body weight and tumor volume were measured on days 1, 3, 5, 9, 11, 13. Tumor volume was calculated using the formula: V = ab^2^/2, where “a” is the tumor dimension at the longest point and “b” is the tumor measurement at the widest point (Yuan et al., 2013; Wang et al., 2015). On day 13, the mice were sacrificed and tumor tissues were carefully removed, cleaned and weighed. The tumor growth inhibition rate was calculated from the tumor weight (TW, g) as: Tumor growth inhibition rate = (TW_PBS_-TW_treatment_)/TW_PBS_ × 100%.

### Immunohistochemical Analysis for CD31, PCNA and TUNEL

Representative tumor sections from each group were fixed in 10% formaldehyde and embedded in paraffin. Paraffin sections were dewaxed in xylene and rehydrated through a graded series of ethanol. After endogenous peroxidase activity was quenched with 3% hydrogen peroxide for 15 min, the sections were treated with normal goat serum for 20 min, then incubated with the primary anti-CD31 (1:200 dilution) or anti-PCNA antibody (1:200 dilution) for 2 h at room temperature and washed with PBS. HRP-labeled anti-goat IgG was applied to sections of CD31 or PCNA for 30 min at 37°C. Color was developed by incubating with DAB and washing with distilled water for 20 min. Sections stained with CD31 were also stained with 1 μg/mL DAPI for 5 min at 37°C. For fluorescent TUNEL assay, after permeabilizing with Triton X-100, the slides were treated with proteinase K for 30 min at 37°C and then incubated in terminal deoxynucleotidyl transferase for 1 h at 37°C in the dark. The slides were also stained with DAPI (1 μg/mL). Images were obtained using an Olympus IX51 fluorescence microscope. Cells and MVD were quantified by measuring pixels in 10 consecutive fields at 40 × magnification. Positive cells were scored manually.

### Statistical Analysis

Values are expressed as means ± SEM from 3 to 6 independent experiments. Two-tailed Student’s *t*-test and one-way ANOVA with Dunnett’s multiple comparison test were used to determine the significance of differences. In any case, *p* < 0.05 was considered as statistically significant. Statistical analysis was assessed using Statistical Package for the Social Sciences (SPSS/PC 20.0, Chicago, IL, United States).

## Results

### N-myristoylation of CM4 Increased the Anticancer Activity in Breast Cancer Cells

**FIGURE 1:**
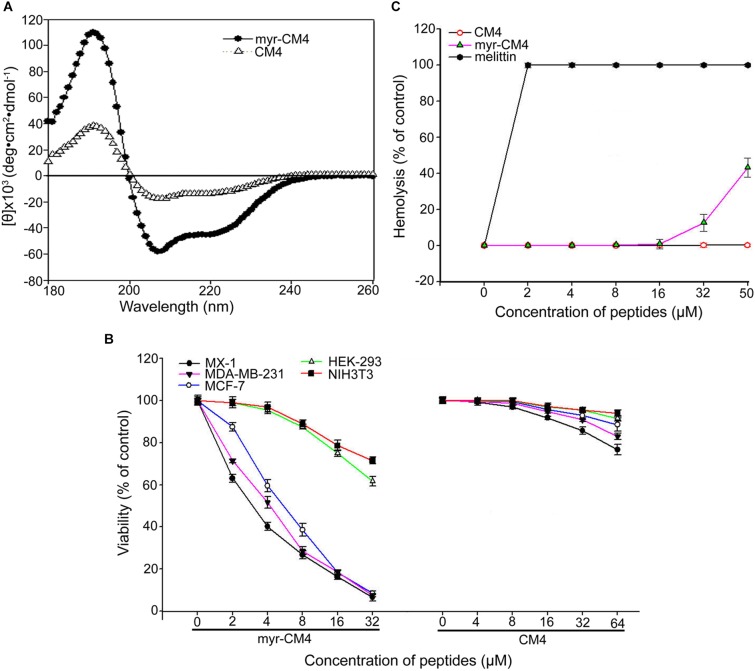
Effects of N-myristoylation of CM4 on the structure and cytotoxicity to breast cancer cells and normal cells. **(A)** Secondary structure analysis by CD spectroscopy. Far-UV CDspectra were conducted at room temperature in TFE/water mixtures at 50% (v/v). Peptide concentration was 250 μg/mL. **(B)** Comparison of the cell viability of three breast cell lines and two normal cell lines after treated by different concentration of myristoyl-CM4 and CM4 by MTT assay. **(C)** Hemolytic activity was tested by mouse erythrocytes. Melittin was used as a control. All assays were performed as described in section 2. Results are mean ± SEM of 4–6 different experiments.

The secondary structure of myristoyl-CM4 was estimated by CD spectroscopy in a solution containing 50% TFE (v/v) (Figure 1A). Both myristoyl-CM4 and CM4 exhibited a positive band at 195 nm and two negative bands at 208 and 222 nm in 50% TFE, suggesting that N-myristoylation did not change the α-helical conformation. CDNN program showed that myristoyl-CM4 presented higher helical content (53.5%) compared with CM4 (30.4%). This implied that N-myristoylation improved the α-helical conformation. The anticancer activity of myristoyl-CM4 against breast cells was assessed using the MTT assay (Figure 1B). A dose-dependent cytotoxic effect on breast cancer cells was observed with myristoyl-CM4 treatment. The IC_50_ of myristoyl-CM4 was 3 μM in MX-1 cells, 4 μM in MDA-MB-231 cells, and 6 μM in MCF-7 cells. However, CM4 displayed low anticancer activity to breast cancer cells, for the cell viability remained above 80% even at the concentration of 64 μM. These data indicated that N-myristoylation greatly enhanced the anticancer activity of CM4. Myristoyl-CM4 also exhibited enhanced cytotoxicity against NIH3T3 and HEK-293 cells. However, at a concentration of 8 μM, myristoyl-CM4 showed little cytotoxicity against NIH3T3 or HEK-293 cells. At a concentration of 32 μM, cell viability remained above 60%. Hemolytic assay showed that myristoyl-CM4 had no hemolytic activity at a concentration of 20 μM (Figure 1C), whereas at 50 μM, approximately 40% hemolysis was observed. These data indicated that N-myristoylation of CM4 enhanced the cytotoxicity of myristoyl-CM4 both against normal cells and breast cancer cells. However, the cytotoxicity on breast cancer cells was greater than that on normal cells, resulting in higher toxicity and specificity toward breast cancer cells.

### N-myristoylation of CM4 Increased the Membrane Affinity and Trans-membrane Activity

**FIGURE 2:**
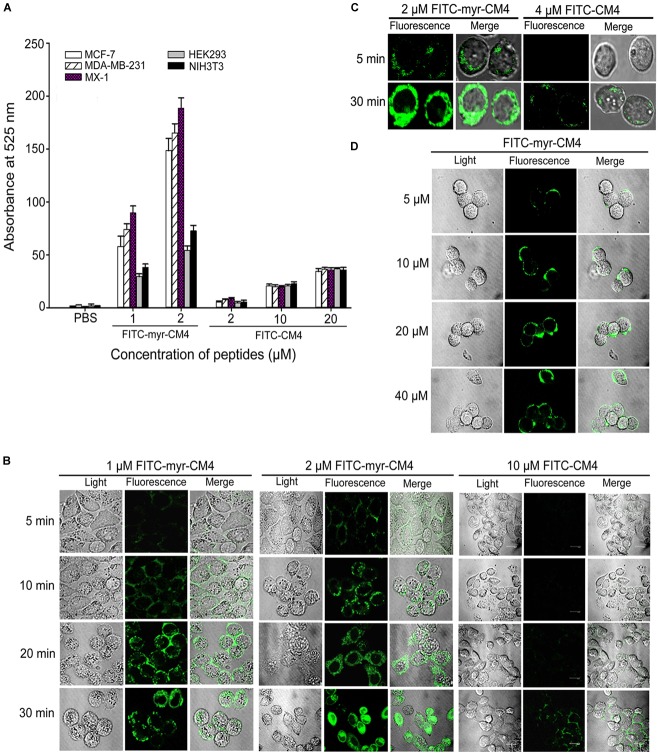
Effects of N-myristoylation of CM4 on the membrane affinity and trans-membrane activities. **(A)** Comparison of fluorescence intensity in five cell lines between myristoyl-CM4 and CM4. Cells were incubated with FITC-labeled peptides for 30 min at 37°C and analyzed by flow cytometry. Results are mean ± SEM of 3 different experiments. **(B)** CLSM images of MCF-7 cells treated with FITC-peptides for different time. **(C)** CLSM images of MX-1 cells treated with FITC-peptides. **(D)** CLSM images of HEK-293 cells treated with FITC-peptides.

To examine whether N-myristoylation had an effect on the interaction between CM4 and the plasma membrane, the membrane affinity was examined in breast cancer cells and normal cells by FITC fluorescence-activated flow cytometry. The data revealed a marked difference between myristoyl-CM4 and CM4. A significant increase in the fluorescence intensity of myristoyl-CM4 was observed compared with that of CM4, indicating that myristoyl-CM4 had a stronger membrane affinity to the cell surface of MX-1, MDA-MB-231, and MCF-7 cells than CM4 (Figure 2A). Compared with FITC-CM4 treatment (2 μM), the FITC fluorescence was 28-fold higher in MCF-7 cells, 15-fold higher in MDA-MB-231 cells, and 22-fold higher in MX-1 cells in response to FITC-myristoyl-CM4 treatment (2 μM). In normal HEK293 and NIH3T3 cells, the fluorescence intensity of FITC-myristoyl-CM4 was only 1/2 to 1/3 of that in MCF-7, MDA-MB-231 and MX-1 breast cancer cells. This implied that the membrane affinity of myristoyl-CM4 to breast cancer cells was higher than that to normal cells.

CLSM was further used to determine the binding and localization. The FITC-myristoyl-CM4 signal was stronger than the FITC-CM4 signal. After incubation for 5 min, the FITC-myristoyl-CM4 signal was detected mainly at the cell surface of MCF-7 cells (Figure 2B) and MX-1 cells (Figure 2C). After incubation for 30 min, the FITC-myristoyl-CM4 signal was detected in the cytoplasm, indicating that myristoyl-CM4 could penetrate into the cells. However, to HEK-293 cells, weak fluorescence of FITC-myristoyl-CM4 was observed on the surface of cells at a concentration of 10 μM (Figure 2D). At 40 μM, FITC-CM4 fluorescence was observed on the surface of HEK-293 cells, whereas no fluorescence was observed in the cytoplasm. These results indicated that N-myristoylation of CM4 increased the membrane affinity in both breast cancer cells and normal cells, and the affinity to breast cancer cells was higher than to normal cells. And N-myristoylation also resulted in the *trans*-membrane transport of myristoyl-CM4 to the cytoplasm of breast cancer cells.

### Glycosylated Molecules Mediated the Interaction Between Myristoyl-CM4 and the Plasma Membrane

**FIGURE 3:**
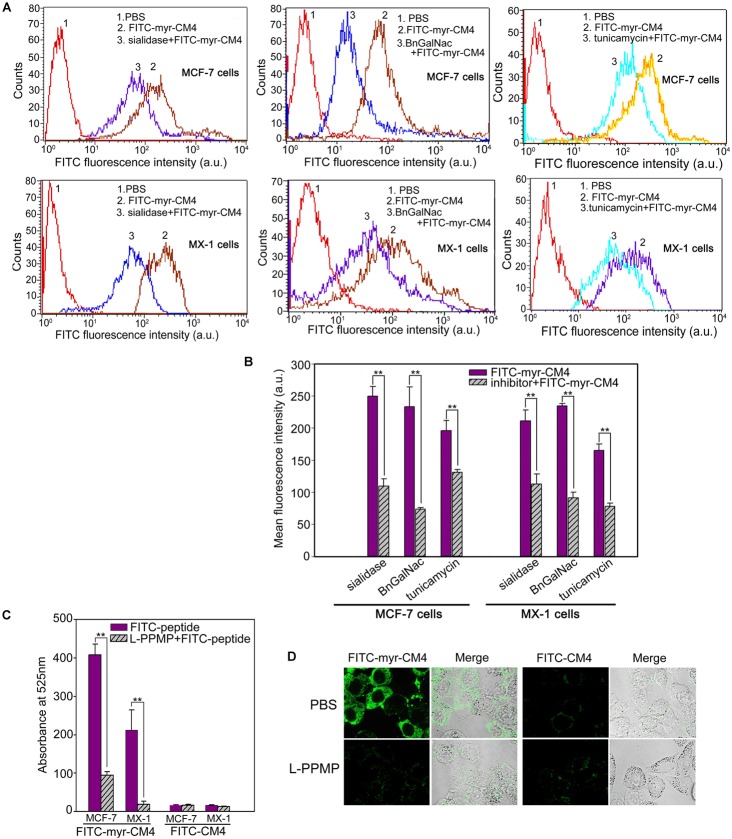
Effects of glycosylated molecules on the membrane affinity of myristoyl-CM4 to breast cancer cells. Cells were pretreated with the following treatments: sialidase (0.1 U/ml, 30 min), BnGalNac (2 mM, 48 h), tunicamycin (3 μg/ml, 24 h) and L-PPMP (2 μM, 48 h). Then cells were incubated with 3 μM FITC-myristoyl-CM4 for 30 min at 37°C in the dark. **(A,C)** FITC fluorescence intensity of MX-1 and MCF-7 cells was analyzed by flow cytometry at 488 nm excitation. **(B)** Reduced fluorescence density was induced by the treatments. **(D)** Images of MCF-7 cells treated by L-PPMP compared FITC-myristoyl-CM4 (3 μM) with FITC-CM4 (3 μM) were acquired by CLSM observation (excitation, 488 nm; emission, 525 nm). Results are mean ± SEM of 3 different experiments, ^∗∗^*p* < 0.01.

Myristoyl-CM4 had higher cytotoxicity and membrane affinity in breast cancer cells than in normal cells. Over-expressed glycosylated molecules play an important role in the binding of unmodified AMPs to breast cancer cells (Han et al., 2013). Sialidase is a specific enzyme that hydrolyses the terminal sialic residues in oligosaccharides, and BnGalNac is a specific inhibitor of *O*-glycosylation. Tunicamycin is a nucleoside antibiotic that blocks the synthesis of all *N*-glycans. Sialidase, BnGalNac or tunicamycin treatments resulted in a significant decrease in FITC fluorescence intensity for FITC-myristoyl-CM4 in both MCF-7 cells and MX-1 cells (Figure 3A). Therefore, glycosylated molecules including sialic acid, *O*-mucin, and *N*-glycans were identified as important targets for myristoyl-CM4 binding to breast cancer cells. The effect was as follows: BnGalNac > sialidase > tunicamycin (Figure 3B). Ganglioside is an important glycolipid in the plasma membrane of breast cancer cells. Treatment with L-PPMP, an inhibitor of ganglioside biosynthesis, resulted in a more than four-fold decrease in FITC fluorescence intensity of FITC-myristoyl-CM4, as indicated by FACS analysis (Figure 3C). These results were further confirmed by confocal microscopy observations (Figure 3D). Therefore, ganglioside also mediated the binding of myristoyl-CM4 to breast cancer cells.

### Myristoyl-CM4 Targeted Mitochondria and Caused Mitochondrial Dysfunction

**FIGURE 4:**
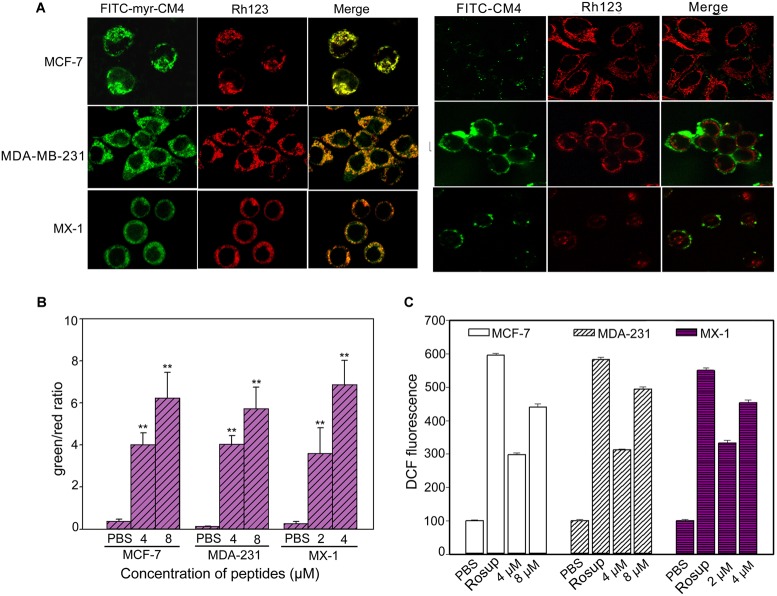
Intracellular effect of myristoyl-CM4 on mitochondria in breast cancer cells. **(A)** Co-localization observation was conducted in MX-1, MCF-7 and MDA-MB-231 cells with 30 nM **Rho123** to label mitochondria for 45 min and then incubated with 2 μM FITC-myristoyl-CM4 for 30 min. **(B)** Mitochondrial depolarization assessed as the fluorescence shift of JC-1 from red to green in MCF-7, MDA-MB-231 and MX-1 cells. After treated with 2, 4, 8 μM myristoyl-CM4 for 16 h respectively, cells were stained with JC-1 and analyzed by flow cytometry at 490 and 525 nm excitation. The emitted light collected at 590 nm (red) and 530 nm (green). **(C)** ROS production measured by flow cytometric analysis using DCFH-DA after different treatments for 10 h. Results are mean ± SEM of 3 different experiments, ^∗∗^*P* < 0.01.

Co-localization of myristoyl-CM4 with mitochondria was detected in the MX-1, MDA-MB-231 and MCF-7 cells (Figure 4A). After being transported to the intracellular compartment, myristoyl-CM4 could target mitochondria. To examine changes in Δψm in response to myristoyl-CM4 treatment, the mitochondrial-specific dye JC-1 was used. JC-1 accumulates in intact mitochondria where it forms aggregates with high Δψm, emitting red fluorescence. When collapses, there is a shift in JC-1 fluorescence from red to green. After exposure to 2 μM myristoyl-CM4 in MX-1 cells, 4 μM myristoyl-CM4 in MCF-7 and MDA-MB-231 cells for 16 h, the green/red ratio increased from 0.27 to 3.59 in MX-1 cells, 0.37 to 4.03 in MCF-7 cells and 0.14 to 4.03 in MDA-MB-231 cells (Figure 4B). These data showed an obvious shift in JC-1 fluorescence from red to green, indicating the disruption of Δψm induced by myristoyl-CM4 treatment.

One of the first events of mitochondrial dysfunction is the generation of ROS. ROS was quantified using DCFH-DA fluorescent dyes (Figure 4C). Compared with the PBS control, 2 and 4 μM myristoyl-CM4 increased the intracellular ROS level by approximately 2.5-fold and 3.6-fold, respectively in MX-1 cells, whereas 4 and 8 μM myristoyl-CM4 increased ROS by approximately 2.5-fold and 4-fold in MDA-MB-231 and MCF-7 cells. These data indicated that myristoyl-CM4 significantly induced ROS generation in the three breast cancer cell lines.

### Myristoyl-CM4 Induced Mitochondria-Dependent Apoptosis

**FIGURE 5:**
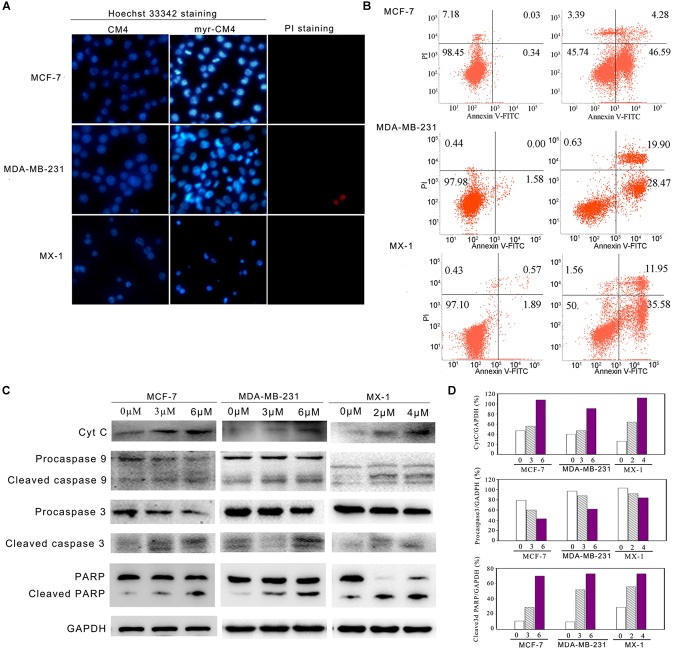
Apoptosis detection in breast cancer cells induced by myristoyl-CM4. **(A)** After treatment by myristoyl-CM4 (4 μM for MCF-7, 6 μM for MDA-MB-231, 3 μM for MX-1) for 16 h, cells were stained by Hoechst 33342/PI and then observed by fluorescence microscopy. **(B)** Cells were stained by Annexin V/PI and then analyzed by flow cytometry. **(C)** Lysates were harvested and the apoptotic proteins (cytochrome c, caspase 9, caspase 3, and PARP) were detected by western blotting. **(D)** The relative amounts of cytochrome c, procaspase 3 and cleaved PARP versus GAPDH were determined by western blotting results and Image J densitometric analysis.

Apoptosis was analyzed using Hoechst 33342/PI double staining. Cells undergoing apoptosis are characterized by chromatin condensation and changes in nuclear morphology, and the blue fluorescence of Hoechst 33342 staining becomes brighter. PI is a red fluorescence dye that selectively stains the necrotic cells. Obvious changes in nuclear morphology and condensed chromosomes were observed after myristoyl-CM4 treatment in MCF-7, MX-1 and MDA-MB-231 cells (Figure 5A). No PI fluorescence was observed after myristoyl-CM4 treatment. This result indicated that myristoyl-CM4 induced apoptosis but not necrosis. Similar results were obtained in the Annexin V/PI assay. Treatment of MCF-7, MDA-MB-231, and MX-1 cells with 4, 6, and 3 μM myristoyl-CM4 respectively, resulted in apoptotic rates of 50.87, 48.37, and 47.53% respectively (Figure 5B). Western blot analysis showed that cytochrome c levels increased after myristoyl-CM4 treatment for 16 h, indicating the release of cytochrome c from mitochondria into the cytosol (Figure 5C). The involvement of the mitochondria-dependent apoptosis pathway in MCF-7, MDA-MB-231, and MX-1 cells was further confirmed by analyzing the levels of cleaved caspase 9, caspase 3, and PARP (Figures 5C,D). Taken together, these results suggest that myristoyl-CM4 could target mitochondria and induce mitochondria-dependent apoptosis in breast cancer cells.

### Effect of Myristoyl-CM4 on MDA-MB-231 Tumors *in vivo*

**FIGURE 6:**
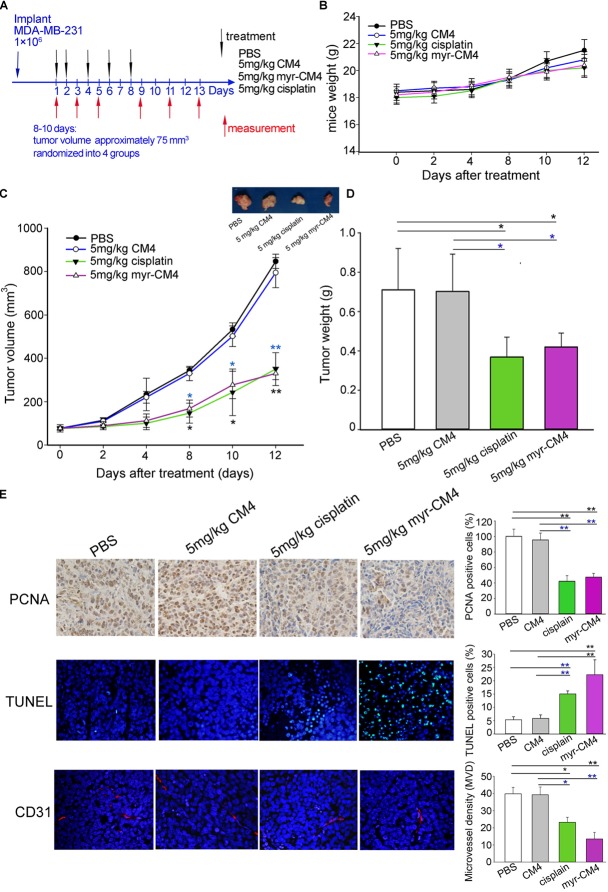
Effect of myristoyl-CM4 on MDA-MB-231 breast xenograft tumors. **(A)** Female BALB/c nude mice were injected subcutaneously right axillary with 1 × 10^6^/mL MDA-MB-231 cells. When tumors reached to about 75 mm^3^, mice were then randomized to four treatment group (*n* = 5) and treated by myristoyl-CM4, cisplatin, CM4 or PBS respectively. Body weight and tumor volume were measured on days 1, 3, 5, 9, 11, 13. **(B)** Mice weight curves over time. **(C)** Tumor growth curves over time. The blue “^∗^” indicated the difference comparation between myristoyl-CM4 group and CM4 group, the black “^∗^” indicated the difference comparation between myristoyl-CM4 with PBS control. **(D)** On day 13, tumors were carefully excised and then measured the tumor weight. **(E)** Paraffin sections were prepared for immunohistochemical staining using anti-CD31 antibody, anti-PCNA antibody and TUNEL staining. Representative images from each group are shown. ^∗^*P* < 0.05, ^∗∗^*P* < 0.01.

Mice were injected subcutaneously right axillary with MDA-MB-231 cells to construct xenograft mice model (Figure 6A). The *in vivo* anticancer activity of myristoyl-CM4 was then assessed for different treatments by injecting through the tail vein of mice for several times. On 12 days after drug treatment, all animals survived the experimental period, and there was no significant loss of body weight in mice treated with myristoyl-CM4 compared with PBS group or CM4 group (Figure 6B). After 8 days of treatments both myristoyl-CM4 treatment (5 mg/kg) and ciaplatin treatment (5 mg/kg) significantly suppressed the growth of xenograft tumors when compared with PBS control or CM4 (5 mg/kg) (*P* < 0.05). On day 12 after treatments, tumor volume between myristoyl-CM4 (or ciaplatin) treatment and PBS (or CM4) treatment showed extremely significant differences (*P* < 0.01) (Figure 6C). There were no significant differences between myristoyl-CM4 group and ciaplatin group, or between PBS group and CM4 group. Myristoyl-CM4 also significantly decreased the tumor weight when compared with PBS control or CM4 treatment (*P* < 0.05) (Figure 6D). The tumor growth inhibition rate of myristoyl-CM4 was 40.85% compared with PBS control. These data indicated that N-myristoylation of CM4 significantly enhanced the depression effect of tumor growth to MDA-MB-231 xenograft tumors mice.

In Figure 6E, the TUNEL assay showed that apoptotic cells were stained green, DAPI (blue) was used to detect live cells. Most cells in sections from tumors treated with 5 mg/kg myristoyl-CM4 were TUNEL-positive, whereas control tumors showed fewer TUNEL-positive cells. Histologic sections were probed with an antibody against CD31, a well-established marker for endothelial cells that is used as an indicator of the degree of vascularization. The results showed decreased numbers of CD31^+^ cells among the tumors treated with 5 mg/kg myristoyl-CM4. Quantification of MVD showed that it was significantly decreased in xenografts treated with myristoyl-CM4 compared with the PBS controls (4.15 ± 0.93 vessels/mm^2^ vs. 14.67 ± 0.95 vessels/mm^2^, *P* < 0.05). PCNA immunofluorescence was used to quantify cell proliferation in tumor sections from all groups. Cell proliferation was significantly inhibited by myristoyl-CM4. Comparing with CM4 treatment, significant increase of TUNEL-positive cells and significant decrease of PCNA-positive cells and MVD were also detected by myristoyl-CM4. These findings indicated that that N-myristoylation of CM4 significantly enhanced the antitumor activity *in vivo in* MDA-MB-231 xenograft. The antitumor activity may resulted from inducing apoptosis or by inhibiting proliferation or vascularization.

## Discussion

Over the past 10 years, several groups reported that the conjugation of fatty acids to AMPs resulted in the enhancement, retention, or loss of antimicrobial activity (Avrahami and Shai, 2004; Chu-Kung et al., 2010; Li et al., 2013). However, there are few reports on the anticancer activity of AMPs conjugated with fatty acids (Sturzu et al., 2009). Our study provides the first evidence of the anticancer effect of N-myristoylation of AMP CM4, which exhibited excellent anticancer activity in MX-1, MDA-MB-231, and MCF-7 breast cancer cells (IC_50_ of 3–6 μM). Regarding the effect on prokaryotic cell membranes, studies show that N-acylation of AMPs increases their affinity and the permeation of the microbial membrane and model membrane by altering the electrostatic and hydrophobic properties (Etzerodt et al., 2011; Romo et al., 2011). We speculated that the enhanced anticancer activity of myristoyl-CM4 may result from increased hydrophobic interaction between the mammalian cell membrane and myristoyl-CM4. Our data showed a marked increase of membrane affinity after myristoyl conjugation, with 28-fold, 15-fold, and 22-fold higher binding capacity of myristoyl-CM4 to MCF-7, MDA-MB-231, and MX-1 breast cancer cells than native CM4, which supports our hypothesis. Myristoyl-CM4 also showed an increased ability to form an α-helical structure in membrane-mimicking solvents, which resulted from the increased helical content of myristoyl-CM4 compared with that of CM4. Indeed, in addition to hydrophobicity, multiple studies demonstrated that helicity plays a crucial role in the activity of AMPs, and the formation of a secondary structure is considered as a driver for peptide insertion into the membrane (Huang et al., 2015). Therefore, the higher hydrophobicity and helicity resulting from N-myristoylation may contribute to a more effective interaction between a peptide and the breast cancer cell membrane, resulting in higher anticancer activity in breast cancer cells.

The effect of N-myristoylation on the selectivity of a peptide is important for its development as an anticancer biotherapeutic. Researchers showed that addition of a fatty acid to an AMP not only modifies the antimicrobial activity, but also changes the selectivity (Avrahami and Shai, 2004; Oh et al., 2004). Normally, increasing the hydrophobicity of AMPs decreases their selectivity for the bacterial membrane, as hydrophobic forces contribute to non-selective binding since the peptide cannot distinguish eukaryotic from prokaryotic cell membranes on the basis of hydrophobicity (Chen et al., 2007; Chu-Kung et al., 2010). However, several studies showed that higher hydrophobicity does not always reduce selectivity (Avrahami and Shai, 2004; Duong et al., 2016). Hydrophobic end modifications such as W- or F-based end-tagged AMPs not only increase antimicrobial potency, but also result in a dramatic improvement in selectivity and low toxicity due to the free energy prize of inserting large W/F-residues in membranes rich in cholesterol (Schmidtchen et al., 2011). A previous study showed that CM4 has no toxicity in normal mammalian cells and erythrocytes (Lu and Chen, 2010). After N-myristoylation, the toxicity of myristoyl-CM4 to normal cells (HEK293, NIH3T3) and erythrocytes both increased together with the anticancer activity. These findings indicated that a higher hydrophobicity increases the cytotoxicity to normal mammalian cells. However, at the IC_50_ concentration in cancer cells, myristoyl-CM4 showed no toxicity to HEK293 and NIH3T3 cells and erythrocytes. At a concentration of 16 μM, more than 80% viability of normal cells and 100% healthy erythrocytes were observed. This indicates that myristoyl-CM4 retains a certain selectivity for cancer cells. Our data showed that the membrane affinity of myristoyl-CM4 toward breast cancer cells was two to three times higher than that toward normal cells. Increased expression of acidic lipids phosphatidylserine, abnormal glycosylation in breast cancer cells, and other factors play important roles in the selectivity of anticancer peptides against cancer cells (Han et al., 2013; Harris et al., 2013). One possible explanation for the selectivity of myristoyl-CM4 is that the net positive charge is the major force for binding to negative molecules on the cell surface even after myristoylation. Therefore, the effect of abnormal glycosylated molecules of breast cancer cells (including *O*-mucin and gangliosides) on the binding ability of myristoyl-CM4 to breast cancer cells was examined. The present binding analysis showed that sialic acid-containing molecules, which are highly expressed in breast cancer cells, were important targets for myristoyl-CM4 binding to breast cancer cells. This implied that, on one hand, hydrophobicity may enhance the interaction between the peptide and the cell membrane, and on the other hand, cationic properties contributed to the selectivity of myristoyl-CM4 for breast cancer cells over normal cells.

The hydrophobicity of α-helical AMPs plays a key role in their interaction with membrane lipids. Peptides with a high hydrophobicity can penetrate deeper into the hydrophobic core of the cell membrane (Chen et al., 2007). Native CM4 was not internalized in breast cancer cells, whereas myristoyl-CM4 could penetrate the plasma membrane and reach the cytosol. This implied that myristoyl was crucial for cellular entry, further highlighting the importance of hydrophobic interactions. A recent study showed that the high hydrophobicity generated from tryptophan end-tagging to the GRR10 peptide efficiently mediated its internalization in melanoma cells (Duong et al., 2016). As demonstrated in the present investigation, myristoyl was efficient in mediating the cellular entry of AMP CM4.

Myristoylation is necessary for the mitochondrial membrane targeting of certain proteins such as Fus1, ctBid, and ChChd (Zha et al., 2000; Darshi et al., 2012; Uno et al., 2015). After internalization, co-localization analysis revealed that myristoyl-CM4 localized to the mitochondria. However, we were unable to demonstrate that this localization was due to the myristoyl segment or the CM4 segment or both. Additional research is needed in the future to clarify the underlying mechanism. Mitochondria are critical mediators of apoptosis and the source of ROS generation. For some AMPs with the ability to enter the cellular space, targeting mitochondria and activating the intrinsic pathways of apoptosis via mitochondrial membrane disruption is a major mechanism of action (Mader et al., 2007; Lee et al., 2008). The present study showed the effect of myristoyl-CM4 on mitochondria and cell death. ROS accumulation increased markedly in breast cancer cells exposed to myristoyl-CM4 and Δψm decreased considerably in MCF-7, MDA-MB-231, and MX-1 cells. Several AMPs such as cecropin A, cecropin 3, and LfcinB-P13 decrease the Δψm and increase ROS generation (Silvestro et al., 1999; Ceron et al., 2010; Pavon et al., 2014). Myristoyl-CM4 also triggered the release of cytochrome c from isolated mitochondria (data not shown), which confirms the effect of myristoyl-CM4 on the permeability of the mitochondrial outer membrane.

Myristoyl-CM4 induced mitochondrial dysfunction by altering the mitochondrial transmembrane potential, increasing ROS generation, and enhancing the permeability of the mitochondrial membrane. Activation of caspase 9, caspase 3, and cleavage of PARP were observed in MX-1, MCF-7, and MDA-MB-231 cells. Myristic acid alone induces apoptosis only at a very high concentration (500 μM) (Beauchamp et al., 2009). These findings suggest that targeting mitochondria and inducing mitochondria-mediated apoptosis is an important anticancer mechanism of myristoyl-CM4. Myristoyl-CM4 also induced apoptosis in MDA-MB-231 xenograft tumors and significantly suppressed the growth of these xenograft tumors. Its antitumor effect at a dose of 5 mg/kg was similar to that of cisplatin at 5 mg/kg, a traditional chemotherapy drug with a number of side-effects. Although great progress has been made in the treatment of breast cancer, in triple negative breast cancer patients, clinical agents are limited by issues such as poor selectivity and the consequent unspecific targeting of healthy mammalian cells leading to deleterious effects together with the development of resistance. In our study, we showed that myristoyl-CM4 possesses excellent anticancer activity in triple negative MDA-MB-231 cells (ER^-^/PR^-^/HER^-^) and MDA-MB-231 xenograft tumors. In addition, myristoyl-CM4 retains a certain selectivity for breast cancer cells. Currently, there is interest in developing more effective and selective anticancer agents for use in breast cancer therapy. AMPs, which are abundant in nature, can be developed as functional anticancer agents. Increasing the positive charge enhances their anticancer activity; however, it decreases the selectivity because of the improved electrostatic interactions (Jiang et al., 2008; Wu et al., 2009). Our current work reveals that increasing hydrophobicity by myristoylation is an effective method to improve the anticancer activity and maintain the selectivity of AMPs.

## Conclusion

**FIGURE 7:**
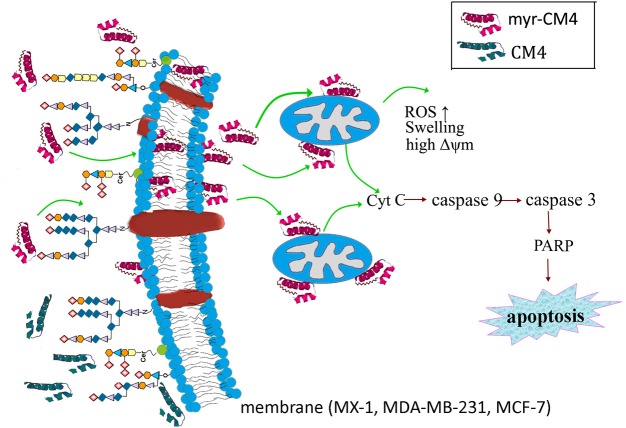
Schematic illustration showing the precise mechanism of myristoyl-CM4 in breast cancer. N-myristoylation significantly increased the membrane affinity toward breast cancer cells and also effectively mediated cellular entry. sialic acid-containing oligosaccharides (including *O*-mucin and gangliosides) were important targets for myristoyl-CM4 binding to breast cancer cells. After internalization, myristoyl-CM4 targeted mitochondria and induced mitochondrial dysfunction, including alterations in mitochondrial transmembrane potential, reactive oxygen species (ROS) generation and cytochrome c release. And then to activate caspase 9, caspase 3 and cleavage of PARP to induce mitochondria-dependent apoptosis in breast cancer cells.

The results presented in the current study demonstrate the precise mechanism of myristoylated CM4 in breast cancer (schematized in Figure 7). In conclusion, conjugation of myristoyl to the N-terminus of CM4 significantly enhances its antitumor activity in breast cancer both *in vitro* and *in vivo*. Myristoyl mediates the interaction between myristoyl-CM4 and the plasma membrane by increasing the binding capacity and cellular entry. After internalization, myristoyl facilitates the interaction between myristoyl-CM4 and the mitochondrial membrane. Targeting mitochondria and inducing mitochondrial dysfunction, and activating mitochondria-dependent apoptosis are the anticancer mechanisms of myristoyl-CM4. Although the toxicity to normal cells and erythrocytes increases with the increase in anticancer activity, myristoyl-CM4 showed higher toxicity and specificity for breast cancer cells. Further effort is needed to increase its selectivity. Taken together, our findings indicate that increasing hydrophobicity by attaching myristoyl would be an effective method for the development of AMPs as mitochondrial-targeting anticancer agents.

## Ethics Statement

All of the animal experiments were approved by the Institutional Animal Care and Use Committee (IACUC) of the Nanjing Normal University and Jiangsu Association for Laboratory Animal Science. We performed all animal work in compliance with the guidelines set forth by the Guide for the care and use of Laboratory Animals.

## Author Contributions

CL and HL performed most of the experiments. TL and YY performed part of the experiments and analyzed the data. XX and SZ analyzed the data and wrote the draft manuscript. HZ and KL revised the submitted version. YC designed the experiments, supported the project, and wrote the final manuscript. All authors read and approved the final manuscript.

## Conflict of Interest Statement

The authors declare that the research was conducted in the absence of any commercial or financial relationships that could be construed as a potential conflict of interest.

## Abbreviations

BnGalNac: Benzyl-2-acetamido-2-deoxy-α-*d*-galactopyranoside
DAB: 3,3-diaminobenzidine
DAPI: 4,6-diamidino-2-phenylindole
DCFH-DA: 2’,7’-dichlorofluorescin-diacetate
DMEM: Dulbecco’s Modified Eagle’s Medium
L-PPMP: Dl-threo-1-Phenyl-2-palmitoylamino-3-morpholino-1-propanol
MTT: 3-(4,5-dimethyl-2-thiazolyl)-2,5-diphenyl-2-H-tetrazolium bromide
MVD: mean vascular density
Rho123: Rhodamine 123
ROS: reactive oxygen species
TFE: 2,2,2-trifluoroethanol
